# Supplementary material for: A novel rapid host cell entry pathway determines the intracellular fate of Staphylococcus aureus
Source: eLife. 2026 Sep 23;13:RP102810. doi: 10.7554/eLife.102810 (PMC13600698; doi:10.7554/eLife.102810)
Supplement: Supplementary file 2 [file elife-102810-fig1-figsupp1-data1.zip › Figure 1-figure supplement 1-source data 1/Figure 1-figure supplement 1-source data 1.pdf]

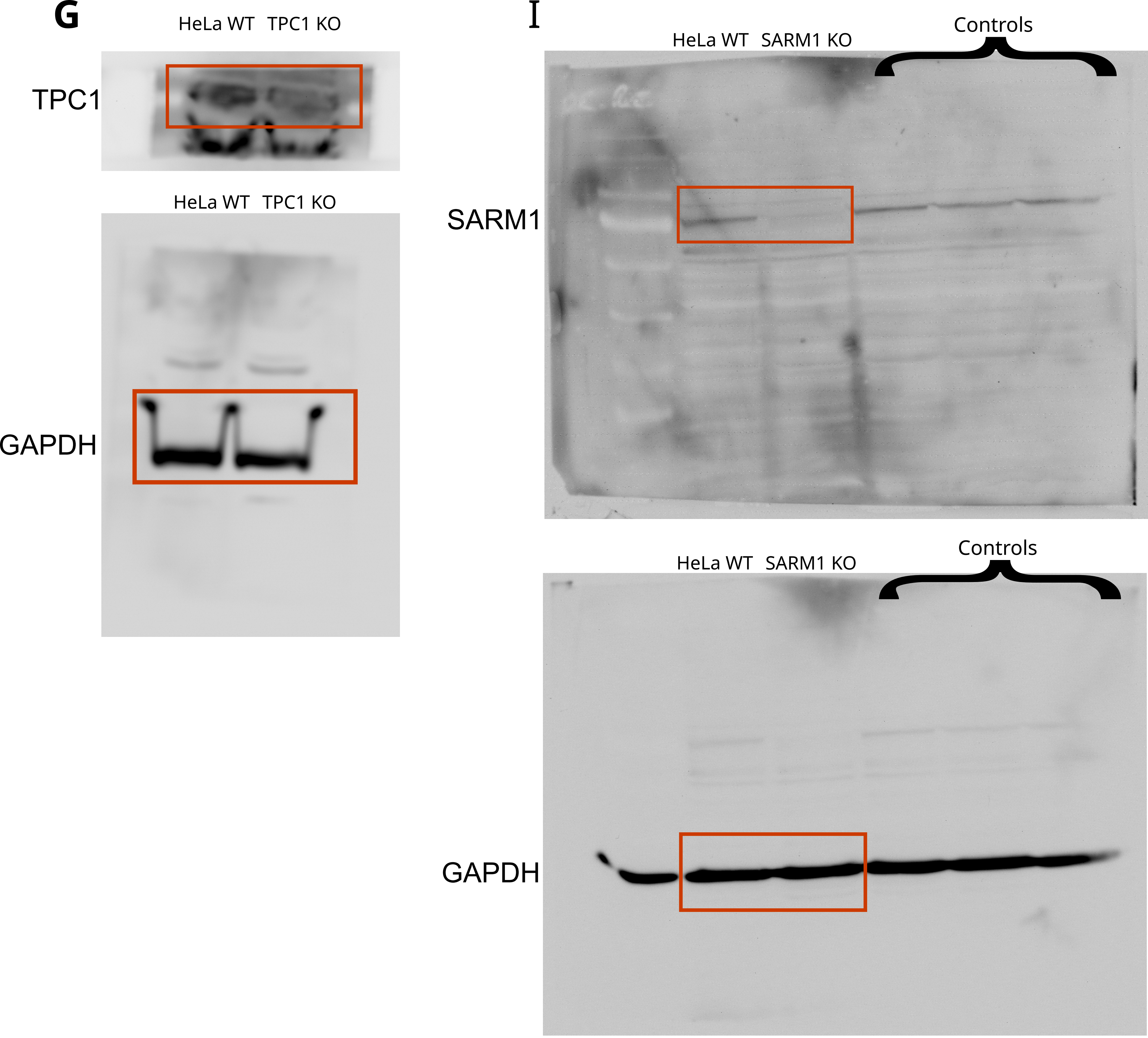

Figure 1-figure supplement 1 source data 1. Original membranes corresponding to Figure 1-figure supplement 1, panels G and I.
(G) Reduction of TPC1 expression in a Cas9-treated cell pool (HeLa TPC1 KO).
(I) Reduction of SARM1 expression in a Cas9-treated cell pool (HeLa SARM1 KO).
TPC1, SARM1 as well as GAPDH (loading control) were detected by Western blots in lysates of wildtype and TPC1 KO or SARM KO cells, respectively
For TPC1 and GAPDH detection the blot membrane was cut and subjected to the different antisera.
