# Supplementary material for: A novel rapid host cell entry pathway determines the intracellular fate of Staphylococcus aureus
Source: eLife. 2026 Sep 23;13:RP102810. doi: 10.7554/eLife.102810 (PMC13600698; doi:10.7554/eLife.102810)
Supplement: Peer review history

# Editor report: eLife Assessment

**DOI:** 10.7554/eLife.102810.5.sa0

**Authors:** Shaeri Mukherjee

**Affiliations:** University of California, San Francisco United States

**Keywords:** Solid, Valuable

This **valuable** study proposes a novel rapid-entry mechanism for *Staphylococcus aureus*, involving the rapid release of calcium from lysosomes. The paper's strength lies in its very interesting hypothesis. The methods used are **solid** and adequately support the conclusions.

# Referee report: Reviewer #2 (Public review)

**DOI:** 10.7554/eLife.102810.5.sa1

[Editors' note: This version was assessed by the editors. The authors have addressed a point raised by Reviewer #2, who thought the authors compared cells grown in low-serum and high serum conditions. This has been clarified in the latest version.]

In the manuscript Ruhling et al propose a rapid uptake pathway that is dependent on lysosomal exocytosis, lysosomal Ca2+ and acid sphingomyelinase, and further suggest that the intracellular trafficking and fate of the pathogen is dictated by the mode of entry. Overall, this is manuscript argues for an important mechanism of a 'rapid' cellular entry pathway of *S. aureus* that is dependent on lysosomal exocytosis and acid sphingomyelinase and links the intracellular fate of bacterium including phagosomal dynamics, cytosolic replication and host cell death to different modes of uptake.

A key strength is the nature of the idea proposed, while continued reliance on inhibitor treatment combined with lack of phenotype / conditional phenotype for genetic knock out is a major weakness.

In the previous version, the authors perform experiments with ASM KO cells to provide genetic evidence of the role for ASM in *S. aureus* entry through lysosomal modulation.

# Author comment: Author response

**DOI:** 10.7554/eLife.102810.5.sa2

**Authors:** Marcel Rühling, Fabio Schmelz, Kim Ulbrich, Fabian Schumacher, Julia Wolf, Maximilian Pfefferle, Magdalena Priester, Adriana Moldovan, Nadine Knoch, Andreas Iwanowitsch, Christian Kappe, Kerstin Paprotka, Burkhard Kleuser, Christoph Arenz, Martin J Fraunholz

**Affiliations:** University of Würzburg Würzburg Germany; University of Würzburg Würzburg Germany; University of Würzburg Würzburg Germany; Freie Universität Berlin Berlin Germany; University of Würzburg Würzburg Germany; University of Würzburg Würzburg Germany; University of Würzburg Würzburg Germany; University of Würzburg Würzburg Germany; University of Würzburg Würzburg Germany; University of Würzburg Würzburg Germany; Humboldt-Universität zu Berlin Berlin Germany; University of Würzburg Würzburg Germany; Freie Universität Berlin Berlin Germany; Humboldt-Universität zu Berlin Berlin Germany; University of Würzburg Würzburg Germany

The following is the authors’ response to the previous reviews.

**Public Reviews:**

**Reviewer #2 (Public review):**

In the manuscript Ruhling et al propose a rapid uptake pathway that is dependent on lysosomal exocytosis, lysosomal Ca2+ and acid sphingomyelinase, and further suggest that the intracellular trafficking and fate of the pathogen is dictated by the mode of entry. Overall, this is manuscript argues for an important mechanism of a 'rapid' cellular entry pathway of *S. aureus* that is dependent on lysosomal exocytosis and acid sphingomyelinase and links the intracellular fate of bacterium including phagosomal dynamics, cytosolic replication and host cell death to different modes of uptake.

Key strength is the nature of the idea proposed, while continued reliance on inhibitor treatment combined with lack of phenotype / conditional phenotype for genetic knock out is a major weakness.

In the revised version, the authors perform experiments with ASM KO cells to provide genetic evidence of the role for ASM in *S. aureus* entry through lysosomal modulation. The key additional experiment is the phenotype of reduced bacterial uptake in low serum, but not in high serum conditions. The authors suggest this could be due to the SM from serum itself affecting the entry. While this explanation is plausible, prolonged exposure of cells to low serum is well documented to alter several cellular functions, particularly in the context of this manuscript, lysosomal positioning, exocytosis and Ca2+ signaling. A better control here could be WT cells grown in low serum.

As the reviewer suggested, we did culture both, WT control cells as well as ASM knock-outs, under low serum conditions before conducting the invasion assays. Hence, the detected effects on *S. aureus* invasion must be caused by lack of functional ASM in the mutant.

We apologize that this did not become evident from the manuscript’s text. We thus included a change in line 259 which now reads:

”To test whether FBS confounded our invasion experiments, we cultivated WT as well as ASM K.O. cells in medium with reduced FBS concentration (1%) and determined the *S. aureus* invasion efficiency (Figure 2I).”

If SM in serum can interfere, why do they see such pronounced phenotype on bacterial entry in WT cells upon chemical inhibition?

We explain the differences between inhibitor-treated WT cells and ASM K.O.s by the severe accumulation of SM upon genetic ablation of ASM. We demonstrated this by HPLC-MS/MS measurements in Figure 2L. If cells were cultured in 10% FBS, an ASM K.O. resulted in approx. 4-times higher levels of cellular SM C18:0 when compared to WT cells, while amitriptyline treatment of WT cells had no effect, and ARC39 treatment increased SM C18:0 levels only by 2-fold. This likely results from different durations of SM accumulation in the cell pools which is caused either by complete absence of ASM (in case of the ASM K.O.) or only in the hour-range upon treatment with the inhibitors.

Under low serum conditions, the severe SM C18:0 accumulation in the ASM K.O. was found decreased (from 4-fold to 2-fold when compared to WT cells; Figure 2M). Here, the WT cells used as reference also were cultured in the same manner as the ASM K.O. A similar pattern was observed for other SM species (Supp. Figure 3). This correlates with the *S. aureus* invasion phenotype in ASM K.O.: under high serum conditions (and resulting in severe SM accumulation) we did not detect an invasion defect, while under low serum conditions (resulting in only moderate SM accumulation) *S. aureus* invasion was reduced in the knock-outs when compared to WT cells cultured in the same conditions, respectively.

While the authors argue a role for undetectable nano-scale Cer platforms on the cell surface caused by ASM activity, results do not rule out a SM independent role in the cellular uptake phenotype of ASM inhibitors.

Since the comments starting with the line above are identical to the previous comments by the reviewer, we assume that these points of criticism still resound with the Reviewer, although we had agreed previously with the reviewer that we do not show formation of ceramide-enriched platforms, we had changed the manuscript accordingly in the previous revision round already (see also our comment below).

The authors have attempted to address many of the points raised in the previous revision. While the new data presented provide partial evidence, the reliance on chemical inhibitors and lack of clear results directly documenting release of lysosomal Ca2+, or single bacterial tracking, or clear distinction between ASM dependent and independent processes dampen the enthusiasm.

We continue to share the reviewer’s desire to discriminate between ASM-dependent and ASMindependent processes, but the simultaneous occurrence of multiple pathways of bacterial uptake is currently the limiting factor and technological challenge in our laboratory, since these events happen rapidly. We do hope that we or others will be able to address these limitations in the future, for instance with the technologies suggested by the reviewer.

I acknowledge the author's argument of different ASM inhibitors showing similar phenotypes across different assays as pointing to a role for ASM, but the lack of phenotype in ASM KO cells is concerning. The author's argument that altered lipid composition in ASM KO cells could be overcoming the ASMmediated infection effects by other ASM-independent mechanisms is speculative, as they acknowledge, and moderates the importance of ASM-dependent pathway. The SM accumulation in ASM KO cells does not distinguish between localized alterations within the cells. If this pathway can be compensated, how central is it likely to be ?

We here want to elaborate again, since our revision experiments demonstrate the ASM-dependency of the rapid uptake under low serum conditions – see also above. We were convinced that the genetic evidence of an *S. aureus* invasion phenotype in ASM K.O.s under these conditions would eliminate the reviewer’s concern about the role of ASM during the bacterial invasion (see also above). Our lipidomics data of ASM K.O.s cultured in 1% and 10% FBS (Figure 2, M, Supp. Figure 3) and inhibitor-treated WT cells (Figure 2L, Supp. Figure 3) show a correlation between SM accumulation and the invasion phenotype observed by us.

We agree with the reviewer, however, that it remains elusive why changes in the sphingolipidome increase ASM-independent *S. aureus* internalization by host cells. One explanation is a dysfunction of the lipid raft-associated protein caveolin-1 upon strong SM accumulation, which was previously shown to appear in ASM-deficient cells (1, 2). A lack of caveolin-1 results in strongly increased host cell entry of *S. aureus* in certain cell types (3, 4). In other cell types, such as A549 cells, *S. aureus* invades in an αtoxin and caveolin-1 dependent fashion (5). It will be interesting to study, to what extent such processes as described by Goldmann and colleagues will depend on ASM. However, a characterization of the mechanism behind these observations requires further experimentation and is beyond the scope of the current manuscript.

As to the centrality of the pathway: we cannot and do not make any assumptions on the centrality of the pathway and its importance in vivo. As scientists we were intrigued by our finding of an ASM dependent uptake pathway for *S. aureus* – especially its speed. In different as of yet still unidentified host cell types or cell lines such a pathway may pose a major entry point for pathogens. Alternatively, we may have identified an ASM-dependent mode of receptor uptake, with which the bacteria “piggyback” into the cells.

The authors allude to lower phagosomal escape rate in ASM KO cells compared to inhibitor treatment, which appears to contradict the notion of uptake and intracellular trafficking phenotype being tightly linked. As they point out, these results might be hard to interpret.

We again want to add that we measured phagosomal escape of *S. aureus* in WT and ASM K.O. cells cultured in 1% FBS (low serum conditions) and compared it to escape rates obtained with host cells cultured in 10% FBS. Again, we infected cells for 10 or 30 min and determined the escape rates 3h p.i. However, the results are similar to escape rates determined with 10% FBS (see Author response image 1). This was addressed already during the manuscript’s first revision. We found that escape rates of *S. aureus* were significantly decreased in absence of ASM regardless of the FBS concentration in the medium.

**Author response image 1.** 

We therefore think that prolonged absence of ASM has additional side effects. For instance, certain endocytic pathways could be up- or down-regulated to adapt for the absence of ASM or could be affected by other changes in the lipidome (that can be minimized but not completely prevented by culturing cells in 1% FBS). This could, for instance, affect maturation of *S. aureus*-containing phagosomes and hence phagosomal escape.

As it is currently unclear in how far the prolonged absence of ASM activity affects cellular processes, we think other experiments investigating the role of ASM-dependent invasion for phagosomal escape are more reliable. Most importantly, bacteria that enter host cell early during infection (and thus, predominantly via the “rapid” ASM-dependent pathway) possess lower phagosomal escape rates than bacteria that entered host cells later during infection (Figure 5, D and E). This is confirmed by higher escapes rates upon blocking ASM-dependent invasion with Vacuolin-1 (Figure 4E) and three different ASM inhibitors (Figure 4C and D). We further demonstrate that sphingomyelin on the plasma membrane during invasion influences phagosomal escape, while sphingomyelin levels in the phagosomal membrane did not change phagosomal escape (Figure5 a and b). This is summarized in Figure 5F.

Could an inducible KD system recapitulate (some of) the phenotype of inhibitor treatment? If *S. aureus* does not escape phagosome in macrophages, could it provide a system to potentially decouple the uptake and intracellular trafficking effects by ASM (or its inhibitor treatment) ?

Knock-downs in our laboratory are based on the vector pLVTHM(6). Inducible knock-downs in the cells would require the introduction of an inducible Tet^on^ system, which the cells currently do not harbor.

However, it needs to be stated that for optimal gene knock-downs, the induction of this system has to be performed by doxycycline supplementation in the medium for 7 days thus leading to several days of growth of the cells, which will allow the cells to adapt their lipid metabolism thus reflecting a situation that we encounter for the K.O.s.

ASM-dependent uptake of *S. aureus* in macrophages has been demonstrated before (7). However, the course of infection in macrophages differs from non-professional phagocytes (8). E.g. in macrophages, *S. aureus* replicates within phagosomes, whereas in non-professional phagocytes replicates in the host cytosol. Absence of ASM therefore may influence the intracellular infection of macrophages with *S. aureus* in a distinct manner.

The role of ASM on cell surface remains unclear. The hypothesis proposed by the authors that the localized generation of Cer on the surface by released ASM leads to generation of Cer-enriched platforms could be plausible, but is not backed by data, technical challenges to visualize these platforms notwithstanding. These results do not rule out possible SM independent effects of ASM on the cell surface, if indeed the role of ASM is confirmed by controlled genetic depletion studies.

We agree with the reviewer that we do not show generation of ceramide-enriched platforms (see also above). We thus already had changed Figure 6F in the revised manuscript to make clear that it remains elusive whether ceramide-enriched platforms are formed. We also had added a sentence to the discussion (line 615) to emphasize that the existence of these microdomains is still debated in lipid research.

We think that the following observations support SM-dependent effects of ASM during *S. aureus* invasion:

(i) Reduced invasion upon removing SM from the plasma membrane (Figure 2N, Supp. Figure 2M)

(ii) Increased invasion in TPC1 and Syt7 K.O. (Figure 2, P) in presence of exogenously added SMase.

However, we agree with the reviewer that we do not directly demonstrate ASM-mediated SM cleavage during *S. aureus* invasion. Hence, we had added a sentence to the discussion that mentions a possible SM-independent role of ASM for invasion (line 556) that reads:

“Since it remains elusive to which extent ASM processes SM on the plasma membrane during *S. aureus* invasion, one may speculate that ASM could also have functions other than SM metabolization during host cell entry of the pathogen. However, we did not detect a direct interaction between *S. aureus* and ASM in an *S. aureus*-host interactome screen (9).”

The reviewer acknowledges technical challenges in directly visualizing lysosomal Ca2+ using the methods outlined. Genetically encoded lysosomal Ca2+ sensor such as Gcamp3-ML1 might provide better ways to directly visualize this during inhibitor treatment, or *S. aureus* infection.

We again thank the reviewer for this suggestion. We already had included the following section in our discussion (then: line 593): “Since fluorescent calcium reporters allow to monitor this process microscopically, future experiments may visualize this process in more detail and contribute to our understanding of the underlying signaling. mechanisms.”

References for the purpose of this response letter:

(1) Rappaport, J., C. Garnacho, and S. Muro, Clathrin-mediated endocytosis is impaired in type AB Niemann-Pick disease model cells and can be restored by ICAM-1-mediated enzyme replacement. Mol Pharm, 2014. 11(8): p. 2887-95.

(2) Rappaport, J., et al., Altered Clathrin-Independent Endocytosis in Type A Niemann-Pick Disease Cells and Rescue by ICAM-1-Targeted Enzyme Delivery. Mol Pharm, 2015. 12(5): p. 1366-76.

(3) Hoffmann, C., et al., Caveolin limits membrane microdomain mobility and integrin-mediated uptake of fibronectin-binding pathogens. J Cell Sci, 2010. 123(Pt 24): p. 4280-91.

(4) Tricou, L.-P., et al., *Staphylococcus aureus* can use an alternative pathway to be internalized by osteoblasts in absence of β1 integrins. Scientific Reports, 2024. 14(1): p. 28643.

(5) Goldmann, O., et al., Alpha-hemolysin promotes internalization of *Staphylococcus aureus* into human lung epithelial cells via caveolin-1- and cholesterol-rich lipid rafts. Cell Mol Life Sci, 2024. 81(1): p. 435.

(6) Wiznerowicz, M. and D. Trono, Conditional suppression of cellular genes: lentivirus vectormediated drug-inducible RNA interference. J Virol, 2003. 77(16): p. 8957-61.

(7) Li, C., et al., Regulation of *Staphylococcus aureus* Infection of Macrophages by CD44, Reactive Oxygen Species, and Acid Sphingomyelinase. Antioxid Redox Signal, 2018. 28(10): p. 916-934.

(8) Moldovan, A. and M.J. Fraunholz, In or out: Phagosomal escape of *Staphylococcus aureus*. Cell Microbiol, 2019. 21(3): p. e12997.

(9) Rühling, M., et al., Identification of the *Staphylococcus aureus* endothelial cell surface interactome by proximity labeling. mBio, 2025. 0(0): p. e03654-24.
